# Global awareness of antigenic peptides – Unraveling the impact on sickle cell anemia patients in the presence of pathogens: A narrative review

**DOI:** 10.1097/MD.0000000000039513

**Published:** 2024-09-06

**Authors:** Emmanuel Ifeanyi Obeagu

**Affiliations:** a Department of Medical Laboratory Science, Kampala International University, Uganda.

**Keywords:** antigenic peptides, global awareness, infection-induced complications, pathogens, sickle cell anemia

## Abstract

Sickle cell anemia (SCA) is a genetic blood disorder characterized by the production of abnormal hemoglobin S (HbS), leading to sickle-shaped red blood cells and various complications, including increased susceptibility to infections. The presence of antigenic peptides, short amino acid sequences derived from pathogens or altered self-proteins, plays a crucial role in immune responses. This review explores the global awareness of antigenic peptides, their role in immune responses in SCA patients, and the challenges and opportunities in managing infections within this vulnerable population. Antigenic peptides are central to the adaptive immune response, facilitating the recognition and elimination of pathogens by T-cells. In SCA, altered antigen presentation and impaired T-cell responses due to chronic inflammation, functional asplenia, and ongoing hemolysis contribute to increased susceptibility to infections. Pathogens such as *Streptococcus pneumoniae* and *Haemophilus influenzae* pose significant risks to SCA patients, highlighting the importance of robust immune responses mediated by antigenic peptides. Strategies such as vaccination and immunotherapy aim to enhance immune function by targeting specific antigenic peptides, thereby reducing infection rates and improving patient outcomes. Advances in genomics and proteomics offer insights into individual variations in antigen presentation and immune responses, guiding the development of tailored therapeutic interventions. Global collaborations are essential to address disparities in healthcare access and implement effective preventive measures, ensuring equitable outcomes for SCA patients worldwide.

## 1. Introduction

Sickle cell anemia (SCA) is a hereditary blood disorder characterized by the presence of abnormal hemoglobin S (HbS), resulting from a point mutation in the β-globin gene. This mutation causes RBCs to become rigid and crescent-shaped, impairing their ability to flow smoothly through blood vessels and leading to various clinical manifestations. SCA primarily affects individuals of African, Mediterranean, Middle Eastern, and Indian descent, with significant global health implications. In addition to chronic hemolytic anemia and vaso-occlusive crises, SCA patients experience heightened susceptibility to infections, which can significantly impact their quality of life and clinical outcomes.^[1]^ The immune system’s response to infections in SCA patients is complex and multifaceted. Central to this response are antigenic peptides – short sequences of amino acids derived from pathogens or altered self-proteins – that are presented on the surface of cells by major histocompatibility complex molecules. These peptides play a crucial role in activating T-cells, which are essential for orchestrating adaptive immune responses against pathogens.^[2]^ Antigenic peptides serve as key mediators in the recognition and clearance of pathogens by the immune system. In SCA patients, the pathophysiological consequences of chronic hemolysis, functional asplenia, and ongoing inflammation create a unique immunological landscape. These factors contribute to altered antigen presentation and impaired T-cell function, compromising the ability of the immune system to effectively respond to pathogens. Consequently, SCA patients are particularly susceptible to infections caused by encapsulated bacteria such as *Streptococcus pneumoniae* and *Haemophilus influenzae*, as well as viral pathogens including influenza and parvovirus B19.^[3]^ The global awareness of antigenic peptides in the context of SCA extends beyond immunological considerations to encompass clinical management and therapeutic strategies. Vaccination against encapsulated bacteria has proven effective in reducing infection rates among SCA patients. However, challenges persist, including vaccine efficacy in the face of altered immune responses and the need for continuous adaptation to evolving pathogen strains. Moreover, advancements in immunotherapy, including peptide-based vaccines and T-cell therapies, offer promising avenues for enhancing immune responses and mitigating infection risks in this vulnerable population.^[4]^

As the global burden of SCA continues to rise, particularly in regions with high prevalence rates, the importance of antigenic peptides in infection management cannot be overstated. International collaborations and research initiatives are essential for advancing our understanding of antigen presentation pathways, immune modulation strategies, and personalized medicine approaches tailored to individual patient profiles. By addressing these complexities, clinicians and researchers can optimize therapeutic interventions, improve clinical outcomes, and ultimately enhance the quality of life for individuals living with SCA worldwide.^[5]^ In this review, we aim to explore the multifaceted role of antigenic peptides in immune responses among SCA patients, emphasizing their implications for infection susceptibility, clinical management, and global health initiatives. By synthesizing current knowledge and highlighting emerging research directions, we seek to foster a deeper understanding of how antigenic peptides influence the pathogenesis of infections in SCA and underscore the urgency of comprehensive strategies to address this critical healthcare challenge. SCA represents a significant public health challenge globally, with approximately 300,000 newborns affected annually worldwide. Beyond its genetic basis and clinical manifestations, the impact of SCA extends to increased vulnerability to infections, particularly among pediatric populations in resource-limited settings. The role of antigenic peptides in mediating immune responses to infectious agents is critical for understanding the pathogenesis of infections in SCA and informing targeted interventions aimed at reducing disease burden and improving patient outcomes.^[6]^

## 2. Aim

This review aims to comprehensively examine the role of antigenic peptides in modulating immune responses and influencing infection susceptibility among individuals with SCA.

### 2.1. Rationale

SCA is a prevalent genetic blood disorder characterized by the production of abnormal hemoglobin S (HbS), leading to significant morbidity and mortality worldwide. One of the hallmark complications of SCA is increased susceptibility to infections, which significantly impacts patient outcomes and quality of life. Understanding the role of antigenic peptides – critical mediators of immune responses – in infection susceptibility among SCA patients is essential for advancing clinical management strategies. Antigenic peptides are short sequences of amino acids derived from pathogens or altered self-proteins that play a pivotal role in initiating and regulating immune responses. In the context of SCA, where chronic hemolysis, functional asplenia, and ongoing inflammation compromise immune function, the dynamics of antigenic peptide presentation and T-cell activation are particularly critical. Altered antigen presentation pathways and impaired T-cell responses contribute to the increased vulnerability of SCA patients to infections, particularly those caused by encapsulated bacterial and viral pathogens. This review aims to provide a comprehensive understanding of how antigenic peptides modulate immune responses and influence infection susceptibility in individuals with SCA. By synthesizing current research findings and exploring gaps in knowledge, the review seeks to identify opportunities for improving clinical outcomes through targeted therapeutic interventions. Furthermore, the review aims to underscore the importance of global health initiatives in addressing disparities in healthcare access and implementing effective infection prevention strategies for SCA patients worldwide. Ultimately, elucidating the role of antigenic peptides in the pathogenesis of infections in SCA is crucial for informing evidence-based practices and guiding future research directions. By enhancing our understanding of antigenic peptide dynamics, this review aims to contribute to the development of personalized medicine approaches tailored to the unique immune profiles of SCA patients, thereby optimizing treatment strategies and improving overall health outcomes in this vulnerable population.

### 2.2. Review methodology

#### 2.2.1. Search strategy

A systematic search strategy was employed to identify relevant literature. Electronic databases such as PubMed, MEDLINE, Scopus, and Web of Science were searched using a combination of keywords including “Sickle Cell Anemia,” “antigenic peptides,” “immune response,” “infection susceptibility,” and related terms. The search was restricted to articles published in English from inception to the present to ensure comprehensive coverage of relevant studies.

#### 2.2.2. Selection criteria

Studies were included based on their relevance to the review’s focus on antigenic peptides and their role in immune responses and infection susceptibility in SCA. Peer-reviewed research articles, systematic reviews, meta-analyses, and clinical trials were considered. Studies that investigated antigen presentation pathways, T-cell activation mechanisms, infection rates, vaccination strategies, immunotherapy approaches, and global health implications in SCA patients were prioritized.

#### 2.2.3. Data extraction and synthesis

Data extraction focused on key findings related to antigenic peptides, immune responses, and infection susceptibility in SCA. Information extracted included study objectives, methodologies, participant characteristics (if applicable), main findings, and implications for clinical practice and research. Data synthesis involved organizing and summarizing extracted information to identify overarching themes, trends, and areas requiring further investigation.

#### 2.2.4. Critical appraisal

The quality and relevance of included studies were critically appraised to ensure rigor and validity. Methodological strengths and limitations, including sample size, study design, potential biases, and generalizability of findings, were considered during data synthesis and interpretation. Studies with robust methodologies and significant contributions to understanding antigenic peptide dynamics in SCA were prioritized in the synthesis process.

## 3. Antigenic peptides in SCA

SCA, a hereditary hemoglobinopathy characterized by the presence of abnormal hemoglobin S (HbS), has been a subject of extensive research. While the genetic underpinnings of this disorder have been well-studied, recent attention has turned toward understanding the role of antigenic peptides in the pathophysiology of SCA. Antigenic peptides in SCA primarily originate from the abnormal hemoglobin S.^[4]^ The distinctive structure of sickle hemoglobin results in the formation of abnormal erythrocytes, leading to increased hemolysis. The breakdown of these abnormal cells releases hemoglobin and associated cellular components, giving rise to a myriad of antigenic peptides. This section explores the mechanisms of antigenic peptide formation, highlighting the pivotal role of abnormal hemoglobin in their genesis. Antigenic peptides in SCA contribute to immunomodulation by activating various components of the immune system.^[5]^ This includes the stimulation of proinflammatory cytokines, adhesion molecules, and chemokines, fostering an inflammatory milieu. The subsequent activation of immune cells, such as macrophages and T lymphocytes, plays a crucial role in perpetuating inflammation. This section provides an in-depth analysis of the immunomodulatory effects induced by antigenic peptides in SCA. The presence of antigenic peptides significantly influences the behavior of red blood cells (RBCs) in SCA. Abnormal hemoglobin and associated peptides can lead to increased RBC adhesion, impaired deformability, and enhanced susceptibility to hemolysis. These alterations in RBC dynamics contribute to vaso-occlusive events, ischemia-reperfusion injury, and the overall clinical manifestations of SCA. This section explores the multifaceted impact of antigenic peptides on the functional aspects of RBCs.

Endothelial dysfunction is a hallmark of SCA, and antigenic peptides play a role in its initiation and progression. The interaction between circulating peptides and the endothelium leads to the activation of endothelial cells and the release of vasoactive mediators. These events contribute to vascular complications, further exacerbating the severity of SCA. This section delves into the mechanisms by which antigenic peptides contribute to endothelial dysfunction in individuals with SCA. The presentation of antigenic peptides in SCA is influenced by both genetic and environmental factors. Genetic modifiers, including co-inherited alpha-thalassemia and other polymorphisms, can impact the quantity and nature of antigenic peptides. Additionally, environmental factors such as oxidative stress and inflammation further contribute to the presentation and immunogenicity of these peptides. This section explores the intricate interplay between genetic and environmental factors in shaping the antigenic peptide landscape in SCA.

## 4. Impact of pathogens on antigenic peptides in SCA

SCA, characterized by the presence of abnormal hemoglobin S (HbS), is not only influenced by intrinsic factors but also by external elements, particularly pathogens. Pathogens, ranging from bacteria to viruses, possess the ability to modulate the presentation of antigenic peptides in SCA.^[6]^ The interaction between infectious agents and the immune system can alter the expression and processing of antigens derived from abnormal hemoglobin. This subsection elucidates the mechanisms through which pathogens influence the presentation of antigenic peptides, potentially exacerbating the immune response in SCA patients. The presence of pathogens in SCA triggers immune activation and inflammatory responses, amplifying the impact of antigenic peptides. Pathogen-associated molecular patterns and antigens from infectious agents stimulate the immune system, leading to a cascade of events that synergistically intensify the immunomodulatory effects induced by antigenic peptides.^[7]^ This part of the review explores the interconnected pathways through which pathogens contribute to immune activation in the context of SCA. Pathogens, particularly those with hemolytic properties, can instigate hemolysis and the release of additional antigens in SCA. The increased antigenic load resulting from pathogen-mediated hemolysis may further exacerbate the complications associated with SCA, contributing to a more severe clinical phenotype. The immune response in SCA patients is influenced not only by the abnormal hemoglobin-derived antigens but also by the concomitant presence of pathogens. Pathogens may contribute synergistically to endothelial dysfunction when coupled with the presence of antigenic peptides in SCA. The combined impact of pathogen-induced inflammation and abnormal hemoglobin-derived antigens can escalate vascular complications. Considering the global prevalence of infectious agents, this section examines the epidemiological aspects of pathogen-induced modulation of antigenic peptides in SCA. Variations in the prevalence of certain pathogens may contribute to geographic differences in the severity of SCA.

## 5. Immunomodulation in SCA

SCA, a hereditary hemoglobinopathy characterized by abnormal hemoglobin S (HbS), extends beyond its genetic roots, involving intricate immunomodulatory processes that significantly contribute to the disease’s clinical manifestations. The inherent chronic inflammation in SCA serves as a pivotal aspect of immunomodulation. Abnormal hemoglobin and the ensuing hemolysis release heme and damage-associated molecular patterns (DAMPs), activating innate immune responses.^[8]^ Pro-inflammatory cytokines, such as interleukin-1β and tumor necrosis factor-α, orchestrate a cascade that perpetuates inflammation. Mononuclear phagocytes, encompassing macrophages and dendritic cells, play a central role in immunomodulation in SCA.^[9]^ The recognition and phagocytosis of damaged RBCs and released hemoglobin-derived peptides activate these cells, triggering the release of inflammatory mediators. The crosstalk between mononuclear phagocytes and the adaptive immune system further shapes the immune response in SCA. The adaptive immune system, including T lymphocytes and B lymphocytes, undergoes significant modulation in the context of SCA.^[10]^Antigenic peptides derived from abnormal hemoglobin and cellular components stimulate T-cell activation, leading to an altered T-cell profile. Dysregulated B cell responses contribute to the production of autoantibodies and immune complexes. Immunomodulation in SCA extends to the vascular endothelium, where the activated immune response contributes to endothelial dysfunction.^[11]^ Endothelial cells, responding to inflammatory signals, upregulate adhesion molecules and release vasoactive substances, exacerbating vaso-occlusive events. The intricate interplay between the immune system and the endothelium contributes to the pathogenesis of SCA. Oxidative stress, a hallmark of SCA, plays a dual role in immunomodulation. On 1 hand, it contributes to the release of DAMPs and the activation of immune responses.^[12]^ On the other hand, the excessive production of reactive oxygen species can impair immune cell function, creating a delicate balance in the immunomodulatory landscape.

## 6. Therapeutic Strategies

SCA represents a global health burden, demanding comprehensive therapeutic strategies that transcend geographical boundaries. Genetic interventions hold promise for altering the course of SCA. Advances in gene therapy, including CRISPR/Cas9 and gene editing technologies, present opportunities for correcting or modulating the abnormal hemoglobin S.^[13]^ This global perspective acknowledges the ongoing research and clinical trials worldwide, aiming to develop accessible and sustainable genetic therapies for individuals with SCA. Recognizing the intricate immunomodulation in SCA, therapeutic strategies aim to mitigate inflammation and modulate immune responses. Antiinflammatory agents, such as corticosteroids, and immunomodulatory drugs are under investigation. This global perspective calls for collaborative research efforts to identify universally effective immunomodulatory interventions and ensure their accessibility in diverse healthcare settings. Given the heightened susceptibility of SCA patients to infections, a global therapeutic approach involves robust infectious disease management. Vaccination programs, antimicrobial prophylaxis, and early intervention protocols are crucial components. International collaboration ensures the dissemination of best practices, fostering a unified global front against infectious threats to individuals with SCA. Hydroxyurea stands as a cornerstone in SCA management, demonstrating efficacy in reducing vaso-occlusive events and improving overall outcomes.^[14]^ This therapy, with its global applicability, serves as a testament to the importance of accessible and cost-effective treatments. A concerted effort is needed to ensure equitable access to hydroxyurea for individuals with SCA worldwide.

Reducing sickle hemoglobin levels through blood transfusions, particularly exchange transfusions, remains a pivotal therapeutic strategy.^[15]^ A global perspective acknowledges the ongoing challenges related to blood availability, safety, and accessibility. Collaborative efforts are essential to enhance blood transfusion programs, ensuring their effectiveness across diverse healthcare infrastructures. SCA is often accompanied by acute and chronic pain, demanding effective pain management strategies.^[16]^ A global perspective recognizes the importance of comprehensive pain management and palliative care, emphasizing the need for culturally sensitive approaches and access to essential pain relief medications worldwide. Global therapeutic strategies extend beyond medical interventions to encompass education and awareness programs. Empowering communities with knowledge about SCA, its management, and the importance of early intervention fosters a proactive approach. This global perspective emphasizes the role of international collaborations in developing culturally relevant educational initiatives. Advancing therapeutic strategies necessitates ongoing research collaborations and participation in clinical trials on a global scale. By pooling resources, expertise, and patient populations from diverse regions, the international community can accelerate the development and validation of novel therapies for SCA.

## 7. Collaborative Initiatives and Global Awareness

SCA poses a global health challenge that requires concerted efforts and collaborative initiatives to raise awareness, foster research, and implement effective strategies. Establishing international research networks facilitates the pooling of expertise, resources, and data to advance our understanding of SCA.^[17]^ Collaborations between researchers, institutions, and organizations on a global scale accelerate the pace of discovery, fostering breakthroughs in genetic, immunological, and therapeutic research. Global awareness of SCA requires culturally sensitive educational campaigns that transcend linguistic, social, and geographical barriers.^[18]^ Collaborative efforts to develop and disseminate educational materials increase public understanding, reduce stigma, and empower diverse communities to make informed decisions regarding SCA prevention, management, and treatment. Collaborative training programs for healthcare professionals ensure a standardized and updated approach to SCA care worldwide. By sharing best practices, treatment protocols, and advancements in patient management, these initiatives contribute to the creation of a global healthcare workforce equipped to address the complexities of SCA. Collaborative patient advocacy networks provide a platform for individuals with SCA and their families to share experiences, access information, and advocate for improved care. These networks amplify the collective voice of the SCA community, influencing policies, raising awareness, and fostering a sense of global solidarity.

Convening global symposia and conferences facilitates the exchange of knowledge, research findings, and innovations in SCA management.^[19]^ Collaborative events bring together researchers, healthcare professionals, policymakers, and patient advocates, fostering cross-disciplinary discussions and forging partnerships to address the multifaceted challenges of SCA. Leveraging telemedicine and telehealth initiatives enables the global dissemination of specialized care and expertise to regions with limited access. Collaborative efforts in implementing telehealth infrastructure empower healthcare professionals to reach and support individuals with SCA, irrespective of their geographical location. Collaborations between governmental bodies, non-governmental organizations (NGOs), and international health agencies strengthen the foundation for global SCA initiatives.^[20–22]^ These partnerships enhance resource mobilization, policy advocacy, and the development of sustainable healthcare frameworks that transcend borders. Establishing research consortia for clinical trials facilitates the evaluation of novel therapeutic interventions on a global scale. Collaborative efforts in conducting multicenter trials with diverse patient populations enhance the generalizability of findings, accelerating the translation of research discoveries into accessible and effective treatments. Collaborative community engagement programs involve local communities in the design and implementation of SCA awareness campaigns and health initiatives. These programs empower individuals with SCA to actively participate in their care, fostering a sense of ownership and promoting sustainable health practices. Incorporating SCA into global health diplomacy efforts encourages policymakers to prioritize the development of inclusive and equitable healthcare policies. Collaborative dialogs between nations and international organizations pave the way for shared strategies, resource allocation, and a unified approach to tackling the global impact of SCA.^[23–30]^

## 8. Recommendations

1. Strengthen International Research Collaborations: Foster collaborative research networks to enhance the global understanding of SCA. Encourage partnerships between institutions, researchers, and organizations to share data, resources, and expertise, accelerating progress in genetic, clinical, and therapeutic research.
2. Develop Cross-Cultural Educational Programs: Design and implement culturally sensitive educational initiatives that cater to diverse communities worldwide. Collaborate with global health organizations, educational institutions, and community leaders to create materials that raise awareness, reduce stigma, and promote informed decision-making regarding SCA prevention and management.
3. Establish Telemedicine Infrastructure: Facilitate collaborative efforts to establish and expand telemedicine infrastructure, particularly in regions with limited access to specialized care. This initiative enhances the reach of healthcare professionals, providing remote support and expertise to individuals with SCA globally.
4. Promote Global Symposia and Conferences: Encourage the organization of international symposia and conferences that bring together stakeholders from diverse fields, including researchers, healthcare professionals, policymakers, and patient advocates. These events foster cross-disciplinary discussions, knowledge exchange, and collaborative efforts to address the complexities of SCA.
5. Advocate for Comprehensive Healthcare Policies: Collaborate with governmental and non-governmental entities to advocate for comprehensive healthcare policies that prioritize SCA prevention, diagnosis, and management. Engage in global health diplomacy to influence policymakers to allocate resources, implement inclusive policies, and address healthcare disparities related to SCA.
6. Facilitate Patient Advocacy Networks: Support and promote the establishment of patient advocacy networks that transcend borders. These networks empower individuals with SCA and their families to share experiences, access information, and collectively advocate for improved care, research, and policy initiatives.
7. Encourage International Training Programs: Collaborate on the development and implementation of international training programs for healthcare professionals. These programs should aim to standardize SCA care practices, disseminate up-to-date knowledge, and enhance the skills of healthcare providers globally.
8. Participate in Research Consortia for Clinical Trials: Actively participate in or support research consortia focused on conducting multicenter clinical trials for SCA. Collaborative efforts in evaluating novel therapeutic interventions with diverse patient populations enhance the generalizability of findings and expedite the translation of research discoveries into accessible treatments.
9. Implement Community Engagement Initiatives: Work collaboratively with local communities to design and implement community engagement initiatives. Involve community leaders, grassroots organizations, and individuals affected by SCA in the planning and execution of awareness campaigns, health education programs, and support services.
10. Invest in Global Health Infrastructure: Collaborate with international organizations, governments, and philanthropic entities to invest in and strengthen global health infrastructure. This includes supporting initiatives to improve healthcare facilities, access to essential medications, and the overall healthcare delivery system for individuals with SCA worldwide.

## 9. Conclusion

The exploration of antigenic peptides and their role in the immune response of SCA patients reveals a complex interplay between chronic inflammation, immune dysregulation, and pathogen exposure. SCA patients face unique challenges in mounting effective immune responses due to the chronic inflammatory environment and the impaired function of antigen-presenting cells. This compromised immune landscape not only makes SCA patients more susceptible to infections but also hinders their ability to respond adequately to pathogens. Increasing global awareness about the specific immunological challenges faced by SCA patients is paramount. Such awareness can drive research and policy efforts towards better healthcare interventions, ultimately leading to improved patient outcomes. Collaborative global efforts are needed to support SCA patients by advancing research, improving clinical practices, and ensuring access to effective treatments.

## Author contributions

**Conceptualization:** Emmanuel Ifeanyi Obeagu.

**Methodology:** Emmanuel Ifeanyi Obeagu.

**Resources:** Emmanuel Ifeanyi Obeagu.

**Supervision:** Emmanuel Ifeanyi Obeagu.

**Validation:** Emmanuel Ifeanyi Obeagu.

**Visualization:** Emmanuel Ifeanyi Obeagu.

**Writing** – **original draft:** Emmanuel Ifeanyi Obeagu.

**Writing** – **review & editing:** Emmanuel Ifeanyi Obeagu.
